# Consumer perceptions of food safety in animal source foods choice and consumption in Nairobi’s informal settlements

**DOI:** 10.1186/s40795-021-00441-3

**Published:** 2021-07-15

**Authors:** Salome A. Bukachi, Mariah Ngutu, Ann W. Muthiru, Aurélia Lépine, Suneetha Kadiyala, Paula Domínguez-Salas

**Affiliations:** 1 Institute of Anthropology, Gender and African Studies, University of Nairobi (UoN), Nairobi, Kenya; 2 Institute for Global Health, University College London (UCL), London, UK; 3 London School of Hygiene and Tropical Medicine (LSHTM), London, UK; 4 International Livestock Research Institute (ILRI), Nairobi, Kenya; 5 Natural Resources Institute, University of Greenwich, London, UK

**Keywords:** Food safety, Animal-source foods (ASFs), Consumer perceptions, Qualitative, Foodborne diseases, Urban informal settlements, Emic perspectives, Malnutrition

## Abstract

**Background:**

Animal-source foods (ASFs) are high-quality nutrient-dense products key to reducing stunting and micronutrient deficiencies. However, their consumption among the poorest households in urban informal settlements is low. Several drivers beyond price, including health considerations have been reported to drive ASF choice and consumption among consumers. This current study explores consumer perceptions of food safety associated with animal source foods (ASFs) consumption in urban informal settlements with a view to unpacking the health considerations driving their choice and consumption.

**Methods:**

Coupled households with children 6–59 months formed the study sample. The Food Environments Working Group (FEWG) Framework of the Agriculture and Nutrition for Health academy (ANH) was used to guide the study which utilized qualitative methods namely, 60 in-depth interviews (IDIs), 19 focus group discussions, and 19 key informant interviews (KIIs) complemented by unstructured observations. Data were transcribed and analysed according to emerging themes.

**Results:**

Consumer perceptions of food safety are driven by concerns about food production, processing, handling, storage and the health risks associated with consumption of the ASFs. For all the ASFs, lack of traceability of source, unhygienic environments in which they were sold and health risks around consuming too much or improperly cooked products were key perceptions from the community. To mitigate against food safety risks, consumers used strategies such as boiling the ASFs, purchasing their products from trusted retailers, avoiding vendors in unhygienic environments and reducing the amount and frequency of consumption of ASFs or totally avoiding their consumption.

These consumer perceptions are increasingly influencing the ASFs choice and consumption in low-income populations besides other drivers. Notably, given limited incomes that influence their purchasing power and the need for nutritious diets that included ASFs, the dilemma of quality vis-a-vis quantity persists and consumers still accessed and consumed these ASF products to supplement their diets.

**Conclusions:**

To enhance food safety for ASFs, as well as assure consumer access to safe ASFs from informal markets, there is need to contextualize the value chain as informed by consumer perceptions on food safety as these influence their ASFs choice and consumption.

## Introduction

Food safety and food and nutrition security are closely linked issues. This is because unsafe food creates a vicious cycle of disease and malnutrition, particularly affecting infants, young children, pregnant women, the elderly, and the sick [1]. In addition to contributing to food and nutrition security, a safe food supply stimulates sustainable development for populations [1]. Food safety is therefore key in the attainment of the sustainable development goals (SDGs) 2, 3 and 6 respectively for the eradication of nutrition and nutrition-associated diseases; to deliver health and clean water and sanitation as a pre-requisite for health [1, 2].;

Foodborne diseases (FBD) include any illness that results from ingesting contaminated food or drinks and is now a major public health concern in developing countries [2, 3]. FBD negatively affects food and nutrition security, introducing additional costs to the food economy and the public health system as it keeps people from working and thriving [1, 2]. Every year, an estimated 600 million – almost 1 in every 10 people around the globe – fall ill after eating contaminated food and 420,000 die annually, resulting in the loss of 33 million years of healthy life (i.e. Disability-adjusted life years or DALYs) [4], a burden comparable to those of major infectious diseases (i.e. malaria, tuberculosis and HIV/AIDS) [3]. The global burden of disease resulting from FBD falls excessively on the populations of low- and middle-income countries of Asia and Africa with children under 5 years affected the most, potentially contributing to child malnutrition rates [1]. The riskiest foods are livestock and fish products and fresh fruits and vegetables contaminated with animal or human waste [5].

More than half of the world’s population now lives in developing countries such as the ones in the African continent [6]. The increased urban population has resulted in the urbanization of poverty, insecurity of tenure, the emergence of informal settlements and informal food markets [6]. This results in the pronounced spatial inequalities being witnessed across the continent including those related to access to safe and nutritious food. Also, the conditions under which the informal food markets sector operates and the clear lack of control raise concerns relating to the safety and quality of food sold [7, 8].

Studies have shown that food safety matters always cause high levels of concern among consumers [2, 9]. Cornelsen et al. [9] in a cross-sectional study on the drivers of animal-source foods (ASFs) in Nairobi’s informal settlements illuminated the need to address food safety issues to contribute to the attainment of higher levels and diversity of ASFs consumption, which in turn could positively affect children’s nutrition and health outcomes. For example, findings from studies done in different countries revealed that food safety was always a concern for consumers and often their single most important concern about food [2, 10, 11].

Concerning consumers responses to food safety concerns, assessments conducted in the context of Rift Valley fever outbreaks in Kenya found that consumers asked to see butchers’ certificates, and demand for ruminant meat dropped as consumers switched to poultry [12]. According to Kavle et al. [13], the declining variety of foods fed to young children in Egypt between 2005 to 2008, and the lack of poultry raised and owned by households in the wake of the Avian Influenza contributed to their stunted growth (Kavle et al., [13]. Similarly, when the African Swine Fever was initially reported by the media in Vietnam, the majority of consumers either stopped eating pork, shifted to chicken, or went to outlets perceived as safer; showing the correlation between consumer food safety concerns and their feeding patterns [10].

According to the World Health Organization (WHO), millions of people are directly affected by FBD every year in the world [14]. The burden of such illnesses is particularly problematic in developing countries [3] with foods most often implicated being the highly nutritious ASFs and fresh vegetables [2, 15]. Unsafe food containing harmful bacteria, viruses, parasites, or chemical substances, causes more than 200 diseases – ranging from diarrhea to cancers [4]**.** The highest known health burden of foodborne disease is caused by parasites, protozoa, bacteria and viruses in ASFs and fresh vegetables. There are also major concerns, but major evidence gaps, on the health impacts of chemical substances, aflatoxins and microbiological contaminants in food [2, 16].

In Kenya, the nationwide food quality and safety systems are legally controlled by various government agencies under different ministries [17]. These agencies are responsible for the surveillance of food safety in the country and aim to disseminate information on the code of hygiene and safe agricultural practices by various stakeholders in the food chains, from the producers to the consumers. Managing food safety has become a challenging task because of fragmented food chains and the lack of enforcement of government regulations [2]. Consequently, Kenya is confronted with a substantial prevalence of food-borne illnesses with over 70% of all episodes of diarrhea being ingestion of contaminated food and water [18]), with limited research to document consumer perceptions and practices concerning ASFs and food safety. To enhance food safety for ASFs, as well as enable consumer assurance in accessing ASFs from informal markets, there’s a need to contextualize the value chain as informed by consumer perspectives. Notably, though, literature that looks into risk perception of hazards, in relation to particular foods such as ASFs, and the concern about food safety among consumers are few [19]. Assessments of food safety and food risks are often laboratory-based from the perspective of the natural sciences limiting consumer insights [7]. Emic perceptions and insights of consumers are driven by their knowledge base as well as social influences of mass media to inform their responses to food including ASFs safety and risks. An individual’s perception of risks is often dependent on several factors, such as how an individual gets and processes information about a particular event, how he/she perceives the level of risk associated with such an event and the personal experience of the risk. Consumer risk assessments depend on the individual’s judgment of the event [20].

This paper presents qualitative findings emerging from a research project of the drivers of ASFs choice and consumption in Kenya, whose overall objective was to investigate the drivers of ASFs choice and consumption within households residing in Nairobi’s informal settlements. The qualitative exploratory study was anchored on the Food Environments Working Group (FEWG) Framework of the Agriculture and Nutrition for Health academy (ANH) that situated the food environment as the interface that mediates the acquisition of foods (ASFs) to people within the wider food system [21]. This current study seeks to illuminate emic insights of consumer perspectives on food safety of ASFs and how these fuels their choice and consumption of ASFs in Nairobi’s informal settlements. The paper thus contributes to the growing body of literature on consumer perceptions and practices in relation to food choice, consumption and safety concerns. This information is necessary to inform policy and implementation towards safe and sustainable food systems to tackle hunger and malnutrition and importantly ensure health and wellbeing.

## Methods

### Study overview

This exploratory qualitative study aimed at understanding consumer’s perceptions of food safety associated with animal source foods (ASFs) consumption and their choice determinants. The study received ethical approval from the International Livestock Research Institute (ILRI) Institutional Research Ethics committee (IREC) number ILRI-IREC 2018/16/1 following the ethical standards. Prior to participation, written informed consent was obtained from all participants, having gone through the study information sheet. The objectives of the study were clearly stated and participation was strictly on a voluntary basis. Participants were informed of foreseeable benefits. Privacy and confidentiality were assured at all times, and participants had the right to withdraw from the study anytime, even after giving their written consent.

### Conceptual framework

The Food Environments Working Group (FEWG) Framework of the Agriculture and Nutrition for Health academy (ANH) informed the study [21]. It places the food environment at the interface between acquisition of foods and the wider food system. The food environment consists of both the external and personal food environment domains. The external food environment includes food availability, prices, vendor and product properties, and marketing regulations within a given context while the personal food environment includes accessibility, affordability, convenience, and desirability at the individual level [21]. Food safety issues are domiciled mainly in the external food safety environment but in close interaction with the personal food environment. All these interact contionously to determine people’s food acquisition and consumption which in turn impact on health and nutrition outcomes.

### Setting and sample

The study was conducted in low-income areas of the peri-urban Dagoretti North and South sub-counties, in Nairobi County, Kenya. Dagoretti North and South sub-counties cover an area of 29.00 and 25.30 Sq. KM each with 5 administrative wards [22]. The two constituencies constitute mainly of a cosmopolitan population, which live in semi-urban regions with a few of the residents of Dagoretti South mainly engaging in subsistence farming while the majority of those in Dagoretti North are mainly practicing small and medium enterprises [22, 23]. The study setting is a relatively stable population, although it receives some migrant population from all regions in the country [22] Participants for the study were purposively sampled from the low-income informal settlements in Kawangware, Uthiru, Ruthimitu, Mutuini, Kabiro and Gatina wards of Dagoretti North and South constituencies. The inclusion criteria included men and women of reproductive age who were in a couple-based family (with spouses living together) and with a child aged 5 years and below. The study sample was limited to coupled households with children under 5 years of age due to the aims of the original study, to inform the intra-spousal household and gender dynamics including agency, bargaining power and decision making in food choice and consumption. They were identified with the help of community health workers from the study area. Study participants were thereafter engaged through information sharing to obtain their written informed consent to voluntarily take part in the IDIs and FGDs. KII participants were purposively sampled from the study area based on their extensive and / technical knowledge on the aspects of health and nutrition.

### Data collection

The study utilized qualitative methods namely, in-depth interviews (IDIs), focus group discussions and key informant interviews (KIIs). These were complemented by unstructured observations, to inform the study aim of exploring the drivers of ASFs choice and consumption. Nineteen FGDs which included participatory exercises were held with 7 groups of men and 12 groups of women each consisting of 12 participants. A total of 60 IDIs comprising of 40 women and 20 men were also held with the sampled members of the community (consumers) to get more in-depth information on their perceptions and behavior in relation to food safety. Nineteen KIIs were conducted with informants sampled purposively from the health and nutrition sector. Interview guides formulated for each method were used to guide the interviews and discussions. The interviews were audio-recorded, with permission from the study participants, using handheld devices, while handwritten notes were also taken during the discussions to complement the audio-recorded data. The audio recordings helped to capture verbatim proceedings of the discussions and were used to generate transcripts that reflected the discussions.

### Data analysis

Data from the FGDs, IDIs and KIIs were transcribed and translated from Kiswahili into English and entered into NVIVO 11 software for coding. To ensure comprehensiveness and reliability in the development of codes, coding and interpretation were done by different team members. At the outset, 3 researchers independently read through the original transcripts to identify codes and emerging themes to inform the development of the codebook. The coding was also done by 3 researchers to enable intercoder reliability. Thereafter 3 additional researchers critiqued and confirmed the findings. The transcripts were analyzed using different steps to thematic analysis: familiarization with data, generating initial codes, selection, review, definition and naming of themes as well as reporting. Members of the research team undertook final checks for consistency on the application of the codes. These codes in turn were grouped into major categories and then into themes representing perceptions of food safety associated with animal source foods (ASFs) consumption and their choice determinants. In presenting the data, relevant verbatim quotes from men, women and key informants were reported in italics to aid interpretation of the data in each theme. Further, the frequencies of the key findings were summarized and linked to the demographic characteristics of the study participants. This is presented in form of tables and figures (bar charts).

## Results

In total 19 FGDs, 60 IDIs and 19 KII were conducted. From the FGDs and IDIs, the mean age of the women was 32.7 years (range 18–73 years). Most of them were casual workers and only five were employed in the government or private sector. The mean age of the male respondents was 34.3 years (range 17–59 years). The majority of the men were also casual workers with only seven employed in the government or private sector. The majority of the respondents (98%) were Christians. The KII participants’ socio-demographics were not collected except for their livelihood activities which included: clinicians and nutritionists, local community leaders including community health volunteers, community health assistants and chief, meat retailers, slaughterhouse manager and meat inspector.

### Food safety perceptions related to ASFs choice and consumption

**Fig. 1:**
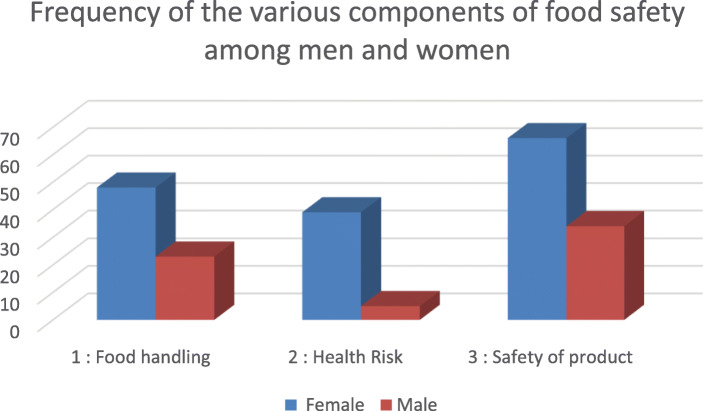
Frequency of the various components of food safety among men and women. Source: FGD & IDI data

Food safety concerns relating to the safety of the products, food handling and health risks were high among both men and women as illustrated in Fig. 1. Men’s main food safety concerns were linked more to safety of the products followed by food handling issues. They were less concerned about the health risks associated with ASF food safety when considering what ASF to consume. On the other hand, women’s concerns cut across the three components, with safety of food being their higher concern. However, much as health risks was third, a higher number of women mentioned it as compared to men. This may be as a result of women being more concerned about the health risks of ASFs on young children.

**Fig. 2:**
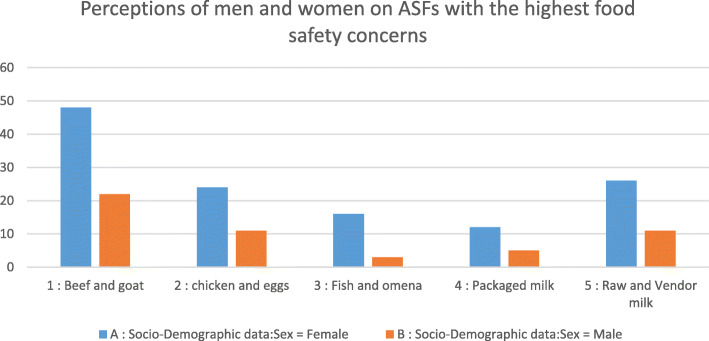
Perceptions of men and women on ASFs with the highest food safety concerns. Source: FGD & IDI data

Community members were increasingly concerned about food safety in relation to ASFs as illustrated in Figs. 1 and 2. While more women were interviewed in this study, both men and women informed the emic perspectives on the health risks of different ASFs. Beef and goat meat were mentioned most frequently signifying the ASFs with highest safety concerns followed by raw and vended milk and closely followed by chicken and eggs as illustrated in Fig. 2. Packaged milk was the ASF with the least food safety concerns. Uncertainties and concerns about the safety of ASFs were largely related to the traceability of the source of these products within the informal settings. Most of the consumers accessed these products from informal markets and vendors and could therefore neither follow the value chain from production to the market hence their uncertainty about the content, quality and safety of the ASFs nor the processes used in their handling, storage and packaging: “*Those ASF foods can be made safer by investigating where they come from and the cleanliness when handling them and their storage too.” (Male KII 018).* Additional concerns were also linked to the fear of possible health risks presented by the continued intake of such foods.

### Perceptions on food safety linked to milk consumption

Milk was the most consumed ASFs in the households, mainly taken in tea for breakfast, as an accompaniment to some meals like *ugali* (stiff porridge) and most importantly, as a core meal for children under 5 years. For milk supply, there were many varieties including raw fresh milk sold by roadside vendors or from milk ATMs (Milk vending/dispensing machines); packaged (fresh) milk from supermarkets or retail shops and processed long-life milk from retail shops or supermarkets. Preference for where to purchase the milk varied across the study participants but was often driven by the concern of the safety of the milk hence opting to go for what was perceived as a safer option. The safety concerns were compressed under broader themes of production, processing and health risks (Table 1).

**Table 1:** Food safety perceptions on milk

ASF	Production	Processing (Handling, storage, packaging)	Health risk
**Milk**	- Unverified source of the raw milk	- Raw milk can be adulterated to increase profit margins for retailers; - Additions of margarine, water, or wheat flour to the milk by retailers to improve the quality of the milk for maximum profits are perceived as adulterants hence contaminating milk; - Containers for dispensing raw milk (ATMs and buckets for roadside vendors) may not be well cleaned and can contaminate the product causing food poisoning	- Not safe for consumption especially by young children who feed on milk mostly within the study setting due to adulteration, chemicals and microbial contaminants which can result in severe disease for children
	For processed / UHT milk the preservatives used in the milk production can be harmful		

**Fig. 3:**
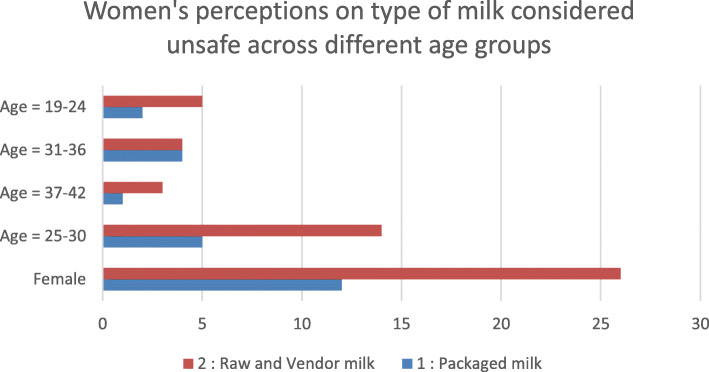
Frequency of women’s food safety perceptions on type of milk across age groups. *Source: FGD & IDI data*

Overall, women were more concerned about the safety of milk especially for feeding young children. Across the different age groups of female respondents, raw/fresh milk was mentioned more frequently as more prone to contaminants and adulteration than packaged milk as illustrated in Fig. 3.

#### Reasons for consumer preference of packaged milk

For participants who preferred packaged milk as a safer option, concerns in relation to the source of product, hygiene, food handling processes as well as adulteration of milk with margarine, water and wheat flour were some of the reasons that were cited for the avoidance of raw and ATM dispensed milk as illustrated in these excerpts:

*“Though the packed one [milk] is a bit more expensive than the fresh one from informal milk vendors, I would rather spend more on the packaged one because of the hygiene issues*.” (Female FGD09)

*“I know that packaged milk contains some chemicals but I prefer it to fresh / raw milk...I consider it as the safest option because as I told you earlier water is added to the raw milk. Not only water but also blue band [margarine]. Milk is also handled by a lot of people, from the one who milks to the different brokers and you never know what happens during the process of transferring the milk*.” (Female IDI018)

#### Reasons for consumer preference of raw milk

Those who preferred raw or ATM dispensed milk cited the presence of chemical preservatives as the main reason for not choosing packaged/processed milk.

*“I do not like the processed/ packaged milk and I also cannot drink it because of the many chemicals so I prefer the raw milk. Even the raw milk we purchase here, it is not that good it is just that you do not have your cow here.” (Male FGD01)*

*The packaged/ processed milk can bring you diseases because it will stay for long without being boiled unlike the fresh/ raw milk that is ATM dispensed , which must be boiled or else it will spoil by morning. That's why I think the ATM dispensed milk is safer and it won't bring diseases and has not been added on preservatives like the long-life/ packaged milk. which will stay for 3 or 4 days because it has preservatives*. (Female FGD04)

#### Perceptions in relation to microbial contaminants

In addition to general concerns about the safety of milk, specific hazards such as microbiological contaminants were discussed. Some participants indicated safety concerns in relation to health risks associated with consumption of milk that may be contaminated with microorganisms and has not been prepared well. “*You know milk can bring about diseases especially if not properly boiled.” (Male IDI21)* Some participants had perceptions about the disease brucellosis and linked it to milk and meat: “*If you have some disease, how is it called? It is a disease that is caused by milk you cannot take milk, even meat sometimes … It is Brucella.”* (Male FGD05). Notably, there was no consensus on whether raw / fresh milk accessed from informal vendors or milk from dispensing machines (ATMs) in shops or the processed packaged long-life milk was better. The raw mik was faulted for possible microbial contaminants and aldulteration linked with food handling and safety of the product. On the other hand, the processed packaged milk was faulted for preservatives that enabled longer shelf life which to the consumers were seen as a health risk factor, since their emic perception on milk was that it was a fresh highly perishable food. Their concerns were that the preservatives added on to the milk to give it a longer shelf life were chemical not good for health and well being. There remains a dilemma in the study population on which milk is safer especially for consumption by young children.

### Perceptions on food safety linked to consumption of meat from cattle and goats

Participants talked of food safety concerns linked to red meat and related by-products like soup, and black pudding (− ‘mutura’_an intestine-encased mixture of minced cow or goat meat, tripe, and cooled blood, flavored with spices). In the meat value chain trust in the food supply channels also represented an important issue, as consumers sighted food source, preparation including hygiene as key factors influencing their food safety considerations as summarized in Table 2 and illustrated in the following excerpt

*“For me, I can talk about meat generally. I am cautious when it comes to buying foods derived from animals from places like kiosks or informal vendors and especially when I am buying food for my children. This is because I lack confidence, to some extent, in such vendors when it comes to matters of hygiene and sources from where they get what they sell. The foods that I have a problem with are beef soup, roasted meat and cooked/fried meat. I prefer when they are prepared at my home rather than when they are prepared by these vendors.” (Male IDI 014)*

**Table 2:** Food safety perceptions on Red meat

	Production	Processing (Handling, storage, packaging)	Health risk
**Red meat**	- Inability to verify the source of the product (including species other than cow, goat, or sheep); - Challenge in verifying that the meat meets veterinary quality checks.	-The presence of many flies may mean the meat is not fresh or can cause food poisoning; - Discoloration on meat eg (Green colour of the meat means that the meat is not fresh);-Cleanliness of the retailer (white clean apron, washing hands after handling money, keeping short nails) and the shop/ butchery denotes good quality products; -Good retailer-consumer relationship is a proxy for good meat quality.	-If eaten in excess can cause diseases like arthritis or cancer.

In addition to concerns about the source and hygiene practices, some participants showed concern about the health risks associated with meat that has not been well prepared: “*… beef has negative effects if you eat a lot of it. If it has not been cooked well it also causes gout,” (Female FGD012).* Meat also be infected with pathogens, *“Meat will give you brucellosis.” (Female FGD 011).*

All the study participants were concerned by the perceived lack of/limited validation of the meat products’ quality by the relevant government bodies. Participants were not sure whether the beef and related products they consumed met the quality standards. They even questioned the inspection role of the concerned departments as they perceived that there were many invalidated reports around the safety and risk of beef as illustrated in the following excerpts:

*We have lost trust in the meat inspection office! Sometimes one is not very sure of the process and criteria used. Sometimes you may find stamped meat in the evening and yet the meat inspector leaves around morning hours after inspection. We are not sure where these other stamps come from.” (Male FGD01)*

*Before I get meat from the slaughterhouse, I make sure that it is stamped. That shows that the meat has been inspected and has not come through the backdoor … you know good quality meat even if you look you just see this is good. … Let me not mention names but there’s meat that is sold without inspection I know of some*. *(Male KII 02)*

### Perceptions on food safety linked to consumption of chicken and eggs

A common belief among the participants was that broiler chicken and eggs were exposed to additives including hormones to boost growth as well as unregulated use of antibiotics for disease management as summarized in Table 3.

**Table 3:** Food safety perceptions on chicken and eggs

	Production	Processing (Handling, storage, packaging)	Health risk
**Chicken and eggs**	- Chicken are injected with antibiotics to fasten maturity; - Broiler eggs are not naturally produced but contain antibiotics and growth boosters, harmful for human health. - The commonly sold ‘*kata kata*’ (non choice cuts of parts of chicken including head, legs, wings, liver, skin, back and necks) may sometimes be meat from wild birds.	- It is difficult to verify the source of the chicken for quality checks, how they are slaughtered and handled.	- Excessive consumption is not good for human health; - In young children, it can cause allergies.

Indigenous eggs and chicken were considered safe and more nutritious yet very expensive for households in low-income settings. Subsequently the consumption of broiler chicken and eggs including chicken cut pieces ‘*katakata’* was more common given the affordability. Consumers were however aware of the food safety related to their consumption. The broiler chicken and eggs were perceived to contribute to the increased cases of non-communicable diseases such as cancer, diabetes and obesity as in the excerpt:

*“… the eggs of recent times are questionable. The chicken has been injected with growth-enhancing hormones so you find the chicken laying three eggs in a day; you find that when you consume them you start getting sick, with diseases like cancer*.” *(Male FGD02)*

*“We have said that we will not give the child soup from the broiler because he or she will have diarrhea because the chickens have been injected.” (*Female FGD04)

In most households, *‘Katakata’* was mostly consumed as it was a cheaper option than a whole chicken. Food safety concerns with ‘*katakata’* were often linked to hygiene, handling and preparation as in the excerpts:

*“The environment in which ‘katakata’ [chicken cut pieces] are prepared is sometimes not clean.” (*Male FGD07)

*“From my perspective, the hygiene and handling of ‘katakata [chicken cut pieces]’ is wanting. You find that the cooking oil used for preparation has been used repeatedly for a week and this can affect you.” (Male KII 06****)***

Inability to confirm the source of the chicken products or even to verify that what they were eating was actually chicken and was also a key safety concern among the participants: *“You might eat it(‘katakata’) only to find out that it is contaminated with something else. For example, a person may be duped to buy chicken meat but instead, it is meat from a wild bird because differentiating the two is difficult especially if you only see the pieces of the meat and not the whole chicken.” (*Male IDI48). Other participants reported being warned against consuming these chicken pieces, *“We were told that ‘katakata’ belongs to some other birds, not chicken, and were advised not to eat them for fear of contracting a disease.” (*Female IDI043).

### Perceptions on food safety linked to consumption of fish

Fish was a delicacy considered as nutritious for children and households in general but was seen as expensive. There were also growing food safety perceptions linked to food handling and safety of the product with the increased demand for the ASF and limited capacity for consumers to verify the source of fish as summarized in Table 4:

**Table 4:** Food safety perceptions on Fish

	Production	Processing (Handling, storage, packaging)	Health risk
**Fish**	- Unverified sources, including whether fish was imported or produced locally; - If imported, the consumer perception is that the product may be genetically modified, and referred to as “plastic fish” hence not safe	- Handling practices: e.g. deep frying -the oil may be recycled and hence not safe; - Most fish is sold by roadside vendors where hygiene and cleanliness is seen as a key concern	-“Plastic fish” may have negative effects on health; - Fish cooked with recycled oil/ fish that is not fresh may cause health problems

Given that within the informal markets the fish were often sourced from roadside vendors, the consumers were concerned about the handling, hygiene and freshness of these ASFs as illustrated in the excerpts:

*“We buy fish from places like Gikomba market (Informal wet market) where you see a lot of flies on those fish, things like those make me wonder about the safety of these foods.” (Male FGD 26)*

Considering that most of these informal markets, are along the roads, are not sheltered, have no canopy and fish-related products are rarely covered, participants indicated that this presents a food safety concern. “*These uncovered foods, like ‘omena’ [small dried fish] sold in the streets along the road gets a lot of dust which may end up contaminating them. The same applies to the big fish. Sometimes you find the oil that they are using to deep fry is dirty because it has been used over and over again. And then I don’t know if you have heard people saying that some use the electricity transformer oil to cook, even here they might be there but we don’t know.” (*Female IDI07).

Additionally, consumers in the informal settings were concerned about the source and quality of fish which was once a preferred ASFs given the notion of prefence of ‘white’ to ‘red meat’ in relation to health. Increasingly, the participants raised concerns about the source of fish, indicating that genetically modified fish is being imported into the country hence can cause disease.

### Mitigating food safety risks

Within the study setting consumers had developed coping measures to tackle their food safety concerns to enable them to complement their diets with ASFs nutrients as summarized in Table 5.

**Table 5:** Consumer perceptions on mitigating ASF food safety risks

Mitigating food safety risks	
Milk	Boiling milk
	For fresh/ raw milk buying from a trusted vendor or directly from a person who has cows and milks for sell
	For packaged milk, only specific brands are perceived as safer, and mostly for making tea not giving to young children.
Beef	Boiling the meat/beef before frying or roasting
	Purchasing from trusted retailers; Checking for a stamp of quality on the meat;
	Avoidance of beef
	Assessment of characteristics such as appearance, color, and smell to get insights into the quality and safety of the ASFs.
Chicken and eggs	Preference for indigenous chicken and eggs over broiler chicken
	Delaying consumption until one can access indigenous chicken from verified sources (Preference given to those from rural-upcountry homes);
	Reducing frequency and amount of ASFs consumed.
Fish	Buying from a trusted retailer;
	Reducing frequency and amount of ASFs consumed;
	Checking for the hygiene of the ASFs by looking at the environment where the fish is sold

The relationship with the retailers or vendors of ASFs was a key coping factor. The participants would rely on a ‘trusted’ vendor from whom they could access quality ASFs products. They talked about their relationship with the butcher as a key factor they often considered as it validated the source and safety of the product. A retailer with whom they have a good relationship was defined as one who meets their expectations for the quality of the ASFs and assured them of fresh, unadulterated products with evidence that meat sold are actually from cattle, sheep, or goats.

*“I trust that specific trader. Once there was a story that, in Kawangware, human meat was being sold in the market so I prefer a specific trusted trader. He usually leaves a piece of hide or skin on the meat for confirmation hence the reason I like it a lot. If it’s a goat, they leave a tail of a goat for confirmation.”* (Female IDI017)

*“I trust him because I know he doesn’t do anything to his meat. He has a lot of customers so he doesn’t need to inject anything into the meat so that it can stay for two or three days. He has a lot of customers so when he brings the meat, it is all bought within a day and a half. He brings new stock every two days.”* (Female IDI035)

Participants reported that they also identified a ‘trusted vendor’, based on hygiene and general presentation of self and product. These, from their perspectives, influenced food quality and safety considerations. Environmental hygiene was key in perceptions of choice of where to purchase ASFs and was seen as a key issue to both women and men as per the following quotes, “*If I look at the butchery and see houseflies on whatever I am going to buy, then I will not buy it*.” *(Female IDI026). You may find that the retailer is selling meat in a dirty place or where there are a lot of flies that will land on the meat and get germs.” (Male IDI019).* Environmental hygiene besides being represented by the vendor’s physical place/site and the display was also extended to the equipment used to contain the ASFs especially milk. *“Cleanliness is key; things like milk require a clean environment. You also need to check … the containers he/she [vendor] is using.” (Male IDI 02).*

The characteristics of the vendor in relation to personal hygiene was also noted and extensively demonstrated in different quotes:

“*When you go to buy meat in a butchery, you can't go to a trader who is just sweating all over and is wearing a jacket that has blood stains all over. He needs to be clean.” (Male FGD03)*

*“The people cutting the meat should ideally put on white coats. They are not supposed to stay with these coats on till evening. Flies will bet following him all over. If they see that it is getting dirty they should change it because they are attending to many people. They should also cut their nails. They should be clean people. If you are clean, then the meat will also be clean. And if you are dirty, flies will be on the meat.” (Female FGD 09)*

Another coping measure was the avoidance of the ASFs including eliminating them from one’s diet or reducing the quantity and frequency of their consumption. In the case of milk, noteworthy was the avoidance of feeding children with processed milk as it was perceived as being harmful to them. This behavior was noted by a majority of the women:

*A small child will have challenges with long life milk because the child is small and that milk has preservatives which can bring problems to the child and that is why we prefer to give fresh milk because it does not have a lot of preservatives that can harm the child unless you contaminate it yourself by the way you handle it and how you boil it. (Female FGD011)*

*Nowadays they use those preservatives in milk so that it can stay for ninety days without going bad. (Female IDI044)*

*For me, I think it is not safe because for one it has chemicals, you can’t keep milk all that time without adding chemicals to it. I cannot give it to my baby because she is still too young to consume those chemicals. I would rather take it myself but not give it to my child, she is still growing.” (Female IDI017)*

For chicken and eggs, broiler chicken and eggs tended to be avoided by most consumers for the preference of indigenous chicken and eggs, this they reported to be mainly fueled by food safety concerns. Indigenous chicken and eggs were perceived to be safer as they are raised more naturally than the improved broiler chicken:

*“The broiler chicken has a lot of chemicals and it is like you are giving someone chemicals, but the indigenous one is left alone to grow naturally, so the eggs from the indigenous ones are tastier than the other eggs.” (Male FGD01)*

*“I will prefer the indigenous eggs because they do not have a lot of chemicals like the grade chickens because we are told those chicken are given a lot of medicine. That is why I think the indigenous one is safer.” (Female FGD 06)*

Processing the ASFs by cleaning and boiling was also another coping measure mentioned to eliminate contaminants or reduce the risks of getting diseases. Washing with water and boiling seemed to be common ways to deal with the risk of getting a disease. For meat-related ASFs, participants indicated, *“… meat, mostly beef, we are told should not be eaten in large quantities because they have some worms and even if we are to eat the meat, we need to boil it thoroughly to remove the worms.” (Male FGD03*). In addition, “*From the butchery, I will boil water and wash the meat with hot water because it has been held by dirty hands. My hands are also dirty*”. *(Female FGD012).* These practices are passed on from one generation to the next especially mother to daughter, *“My mother used to say that if meat doesn’t cook well, it will bring diseases, though I don’t know which disease. So meat has to boil first so that it does not bring diseases.” (Female IDI044).*

With regards to milk, boiling was a common way to eliminate contaminants or reduce the risks of getting diseases:

*“You boil milk because when you boil you kill the germs. Maybe the cow you are getting milk from has a certain disease on the breast and it has affected the udder, you will milk it and then make sure you boil milk.” (Female FGD06)*

*“We are always told that you must boil milk thoroughly because it has so many diseases, the germs in the milk do not die easily.” (Female IDI036)*

Besides boiling the raw milk, packaged milk was also boiled severally with the view to eliminate the chemicals used in their preservation as elucidated in the following excerpt:

*“I usually don’t boil packed [long life] milk first. I allow the water to boil, after boiling I add the milk to the water then let the mixture boil further, when it’s about to spill over, I reduce the flame of the stove, then allow it to boil again, the second time when it’s about to spill over that is when I remove it. The first time I believe that the chemicals in the milk have not been eliminated, so I have to boil it twice.* (Male IDI 04)

### Food safety vs food quality and quantity

Notably, however, despite the food safety concerns raised by the consumers they still accessed and consumed these products. The low economic status of most of these participants seemed to drive their purchasing power and influenced their choice and consumption of ASFs within their informal settings. Although they were often aware of the risk of consuming some foods given the lack of validation for quality and safety, they still consumed these as they were affordable and easily accessible to supplement their food and nutrition needs as well as give, especially, their children balanced diets:

*“If I had enough money I would also buy foods from a different place since I do not trust what we eat in Kawangware.” (Male FGD 05)*

*On my side food is any kind of substance that can keep the day going. Sometimes it can make you satisfied but not all times. This is especially when you have a large family to provide for. It also does not matter if the food is healthy or not as long as you get to live to fight for another day.” (Male FGD).*

The participants wished that the government would look into the issue of food safety and perhaps enforce some regulations to safeguard their wellbeing. The need for government enforcement of the food safety measures for ASFs especially in informal markets to trace and validate the ASFs value chain from production to consumption is necessary as in the key informant excerpt:

*“Before I get meat from the slaughterhouse, I make sure that it is stamped. That shows that the meat has been inspected and has not come through the backdoor … . Depending on how I have learnt the market there are many such cases especially in … let me just not mention names but there’s meat that is sold without inspection I know some.” (Male KII 02)*

## Discussion

Participants articulated concerns about ASFs; particularly the concern that ASFs may not be verified for quality and source and may consist of contaminants, pathogens, or chemical preservatives that bring out health safety dilemmas. Notably as at the time of the study, within the research setting and generally in Nairobi there were ongoing mass media reports and documentaries around the safety concerns of beef particularly highlighting the likelihood of compromised sources of the meat because of laxity in vetting by the government departments concerned [24]. While these reports remained unvalidated, they largely influenced consumer perceptions and concerns about the source, processing and possible health risk of beef products. Media communication plays a vital role in food safety governance [25]. Consumers cited lapses in the food inspection and certification especially of beef but also the unregulated sale of milk as a contributor to food safety issues. This is a key concern that also involves ethics and is present in many developing countries and especially in the informal sector and has been linked to risks of zoonotic diseases [10, 26].

Concerns of consumers in this current study about lack of traceability of the ASFs products back to the production node is a warranted concern and confirm findings in several studies [27, 28] that stress the importance of and need to trace the sources of our food products. However, implementing a full traceability system in the value chains is a challenge to most countries especially developing countries, yet its understanding is critical in developing and implementing appropriate technological interventions suitable for consumer demands [29]. Interestingly also while there were consumer perceptions on food safety, the need to access the nutrition value of ASFs often informed the decision to access the ASFs. While there were perceptions and concerns about food safety, it did not alter dietary patterns as some of the consumers were certain of their ability to avoid safety risks as they trusted their risk mitigation strategies, including vendor selection and safe food handling practices at home. This finding is similar to that of Wertheim-Heck et al., [30] that showed that although Vietnamese consumers were anxious about the food safety of the foods they consumed, consumers generally believed that they were able to minimize those risks by choosing specific outlets to buy different types of foods.

To mitigate the food safety risks in the ASFs especially milk, consumers generally boiled it before consumption. These study findings conform to a previous study that indicated that boiling is key to reducing the pathogens found in milk [19]. However, other quality and safety issues e.g. milk adulteration with water, the addition of antibiotics or other chemical preservatives, aflatoxin contamination persist even when milk is boiled before consumption and storage hence the need for additional safety measures [19, 31, 32].

Most of the eggs, fish, meat and milk sold to the poor in urban Africa are sourced from informal markets (FAO, 2003b). These ASFs are readily available within the informal markets as in the study setting. For example, in countries like Kenya, Mali and Uganda, 80–90% of raw milk is purchased from vendors or small-scale retailers in informal markets [33]. Informal economies have long been the linchpin of food security for both the rural and urban poor in developing countries [34] and informal food markets and street vendors thus play a vital role in the livelihoods and the nutrition security of the poor [7, 35]. In Nairobi, for example, 63–70% of people live in informal settlements [36] and occupy only 6% of urban land often accessing food from informal markets [6]. The conditions under which the informal food markets sector operates raise concerns relating to the safety and quality of food sold [7]. There has always been a complex issue of food handling especially in the mass production of food hence the possibility of food contamination.

Avoidance of consuming ASFs as a mitigating strategy to food safety hazards is not a permanent solution for consumers and may trigger other health conditions, especially among young children and women. ASFs in general contain the highest amount of protein per unit of energy, and protein derived from animal foods is considered the best quality protein, providing all the dietary essential amino acids in adequate proportions hence are important in the growth and development of children [26]. Adding even small amounts of animal products to a plant-based diet can yield large improvements in maternal health and child development, along with many other positive health implications and aid in combating malnutrition [37].

Personal hygiene including donning clean clothing while selling was considered important in this current study in eliciting consumer trust in the safety of the vended ASFs. Indeed, Birgen et al., [7] in their study on microbial contamination of street-vended chicken in Kenya noted that lack of good personal hygiene by vendors can contribute to cross-contamination hence posing a health risk to the consumers of chicken purchased in the open-air markets along the roads. Consumer’s deductions of compromised safety of the ASFs sold by vendors if their environmental hygiene was not clean, and was infestation with flies, are not new. Findings by Chioma et al., [38] and Birgen [7] attributed food safety concerns to unhygienic environments. Improper handling of raw ASFs especially raw meats, presence of flies, unclean vending places, appropriate clean clothing, concerns raised in this current study has been noted in other studies to be positive predictors of *Salmonella spp.*, *E. coli, Staphylococcus* and *C. jejuni* contamination of ASFs [7, 39]. This poses a great concern on the quality and safety of foods purchased from such vendors who play an important role in meeting the food demands of urban dwellers.

Use of the physical attributes based on our senses, such as smell and sight to navigate around the purchase of perceived safe ASFs, have been documented previously [35] as a first step in the process of assessing the quality and freshness of ASFs and fresh fruit juices sold by street venders. However, as much as physical attributes can be a first step in assessing the quality and safety of ASFs, they are not sufficient on their own to determine the safety of foods given that there could be several microbial and chemical contaminants present in foods but not physically visible on a physical assessment. Microbial and chemical agents are great threats to food safety today given the production systems adopted to increase the production of animals to meet the ever-growing food demand of the increasing populations. Consumers reported more on their growing concern about chemicals and antibiotics used in animal production as well as preservatives used in the processing of ASFs including milk. They attributed the rise in non-communicable diseases in Kenya to the use of chemicals and preservatives in food production and processing. However, Birgen et al., [7] and Grace et al. [40] indicate that the increasing threats to food safety in SSA are more microbial rather than chemical hazards. The finding from this current study may mean that consumers in low-income settlements are increasingly getting more aware and concerned about food safety hazards just like their counterparts who shop at supermarkets and specialty stores [40].

Rarely were zoonotic diseases mentioned by the consumers. It’s only in two FGDs were brucellosis was mentioned as a concern yet zoonotic bacterial diseases are increasingly becoming a public health concern and are estimated to be the leading cause of human illness worldwide with a great burden in developing countries resulting in huge economic losses in addition to public health issues [41, 42]. This calls for consumer education on food safety and health risks posed by zoonotic diseases.

The most visible activities in the informal markets are food production (urban, peri-urban and rural), processing, catering and transport, and retail sale of fresh or prepared products (e.g. street food), [43].

Food vending by small and medium-sized enterprises is a common practice along the food chain. This informal segment in the food industry contributes to about 80% of the food products supplies in the market though under very limited hygiene standards [7]. This points to an apparent indication of the gaps in the food safety control mechanisms in the country as is also highlighted in this current study and raises the inadequacies in efforts in coordination of the various agencies involved in maintaining food safety along the food chain in the urban informal settings and markets. In these informal markets, there is usually a longer value chain process from production to distribution causing the challenge with traceability to ascertain the source and quality of the ASFs [2, 33]. These urban diets are dependent on local food systems, from which they need to obtain diverse and healthy foods. There is therefore the need to utilize existing policy frameworks to enable actors from producers to consumers including distributors of the ASFs value chains in different geographical and socio-economic contexts to align to food safety standards and allow for supply and consumption of these to promote nutrition outcomes of young children in urban resource-limited informal settings. More affordable nutrient-rich foods, better nutrition knowledge, trustworthy food hygiene and safety information and practices and policies that promote these factors can leverage nutrition and enable food security and promote sustainable development.

Food producers, retailers and relevant food surveillance authorities in Kenya need to understand consumers’ perspectives on food safety. A better insight into the determinants of consumer concern about food safety will assist policymakers to reduce food safety issues. Notably, some perceptions are misconceptions in relation to ASFs and food safety and these exist as detailed in the results of this study such as, the preservation methods (i.e. long-life milk is not the result of adding chemicals but of killing pathogens and other food spoilers through heat treatment), which might be good to address through relevant marketing campaigns. Also clear is the distrust in government food quality and safety control system which might also need addressing. These will better inform consumer perceptions and address misconceptions on ASFs food safety. Further, it will inform their nutrition decisions as they access safe and affordable ASFs to supplement household diets, especially of children under 5 and tackle malnutrition. The results of this qualitative study complement the quantitative results from the DFC project within which this study was conducted. The quantitative results to be published shortly by this group of authors will expand on some of the relevant issues such as the key importance of trust and perceived meat safety in the choice of retailers.

### Limitations

This study provides in-depth insights into the emic perceptions on food safety in relation to ASFs. It is worth noting that these perceptions on food safety emerged from findings of the broader project study which sought to establish the drivers of choice and consumption of ASFs in an informal setting. Furthermore, the study sample was only limited to coupled households with children under 5 years of age due to the aims of the original study, to inform the intra-spousal household and gender dynamics including agency, bargaining power and decision making in food choice and consumption. Therefore, the perceptions on food safety presented in this paper are not fully exhausted and representative, having only captured a small segement of the population. Further research that explicitly seeks to assess consumer perceptions on a larger scale and across all segements of the population in in low resource settings is recommended. This notwithstanding, the findings of this study are indicative of consumer perspectives on food safety and provide useful information that can be built upon to help mitigate food safety concerns in the community.

The qualitative findings were analyzed using an inductive approach in the interpretation of results hence a limitation. However, the data and subsequent results were analyzed by multiple researchers. At the outset, three researchers independently read through the original transcripts to identify codes and emerging themes to inform the development of the codebook. Coding was also done by three researchers to enable intercoder reliability. Thereafter three additional researchers critiqued and confirmed the findings. This collaborative analysis and writing process increases the validity of these results. Additionally, triangulation of findings from the different qualitative methods of data collection further confirmed our results.

## Conclusions

The study findings describe emic perspectives highlighting food safety concerns. Consumer perceptions and interest in food safety are evolving with more awareness at present. Notably, though there are still gaps in education to allow for better knowledge and understanding of food safety to inform consumer choices and consumption. These were often founded on the limited knowledge by consumers to validate the source of the food and its handling along the value chain from production to consumption, and a lack of trust of both the vendors and the source of the product. Community perceptions on the food safety of ASFs accessed in the informal markets were also driven by a lack of clarity of the food handling processes and the product content that may pose health risks when consumed. Risk assessments and studies seeking to understand consumers’ attitudes, perceptions and knowledge about ASFs safety in Nairobi’s informal settlements is a fundamental step towards ensuring that policymakers are provided with the necessary information to develop and implement policies that protect consumer health. It also provides valuable information for the ASFs value chain actors and stakeholders to improve their practices and provide quality and safe food. These findings lay the foundation upon which future research agenda on urban food safety can be anchored.

## Recommendations

Government effort in addressing food safety and quality of ASFs is important. Institution and enforcement of food quality and safety regulations not only among vendors in informal settlements but across the whole food value chain is critical in dealing with the food safety hazards. Consumer sensitization and education are important in addressing misconceptions around food safety. It will help address consumer perceptions and inform their nutrition decisions as they access safe and affordable ASFs to supplement household diets, especially for children under 5 and help tackle malnutrition.

## Abbreviations

ANH: Agriculture and Nutrition for Health academy
ASFs: Animal Source Foods
DALYs: Disability Adjusted Life Years
ILRI-IREC: International Livestock Research Institute (ILRI) Institutional Research Ethics committee
FBD: Foodborne Diseases
FEWG: Food Environment Working Group
FGDs: Focus group discussionsIDIs- In-depth interviews
KIIs: Key informant interviews
SDGs: Sustainable Development Goals
